# Design and Evaluation of Alginate-Based Hydrogels for Controlled Release of Cationic and Anionic Model Compounds

**DOI:** 10.3390/gels12050407

**Published:** 2026-05-08

**Authors:** Archana Mishra, Kitz Paul D. Marco, Madeleine M. Melancon, Dominic Karl M. Bolinas, Allan John R. Barcena, Marvin R. Bernardino, Marites P. Melancon

**Affiliations:** 1 Department of Interventional Radiology, The University of Texas MD Anderson Cancer Center, Houston, TX 77030, USA; archanamishra56@gmail.com (A.M.); kdmarco@mdanderson.org (K.P.D.M.); s1918479@online.houstonisd.org (M.M.M.); dmbolinas@up.edu.ph (D.K.M.B.); ajbarcena@mdanderson.org (A.J.R.B.); mrpbernardino88@gmail.com (M.R.B.); 2 College of Medicine, University of the Philippines Manila, Manila 1000, Philippines; 3 UTHealth Graduate School of Biomedical Sciences, The University of Texas MD Anderson Cancer Center, Houston, TX 77030, USA

**Keywords:** hydrogel, alginate, controlled release, polyelectrolyte, rhodamine B, trypan blue

## Abstract

Alginate-based hydrogels are well-known materials for drug delivery. However, their sensitivity towards chelators and burst release remain concerns. Polyelectrolyte coatings have been proposed to control drug release from hydrogels, but it remains unclear how the types of coating influence the release kinetics of drugs with varying ionic properties. Hence, we explored combinations of natural (chitosan, alginate) and synthetic (polyethyleneimine [PEI], polyacrylic acid [PAA]) polyelectrolytes to modulate the release kinetics of alginate-based hydrogel beads. Rhodamine B (RhB, cationic) and trypan blue (TB, anionic) were used as model drugs. PAA resulted in a less porous coating, and PEI effectively reduced swelling. While uncoated beads showed burst release, polyelectrolyte coatings slowed release to varying degrees. Chitosan+alginate and chitosan+PAA coatings showed limited impact on RhB release. In contrast, PEI+alginate and PEI+PAA coatings significantly reduced RhB release from 86% (uncoated) to 61% and 6% after 4 days, respectively. Alginate–TB displayed inherently slower release across all systems, with polymer coatings further reducing cumulative release from 49% (uncoated) to 7% in PEI+PAA-coated beads. Overall, polyelectrolyte coatings had a greater influence on the anionic payload, and PEI+PAA-coated beads emerged as promising carriers for controlled release of both cationic and anionic drugs.

## 1. Introduction

Hydrogels have been widely explored as platforms for controlled drug delivery, tissue engineering, and biomedical applications [1,2]. Sodium alginate(SA)-based hydrogels have particularly attracted significant attention due to their mild gelation conditions, biocompatibility, and suitability for encapsulation of sensitive therapeutic agents, including dyes, proteins, and drugs [3,4,5,6,7]. However, the sensitivity of alginate hydrogels to phosphate and citrate ions, along with burst release of entrapped drugs, remains a significant limitation.

Controlled release from hydrogel systems is governed by multiple factors, including polymer composition, crosslinking density, network porosity, swelling behavior, and the physicochemical properties of the encapsulated compound [8]. Polyelectrolyte coating, wherein a core particle is coated with alternating layers of polycations and polyanions, has emerged as a simple and cost-effective strategy to control drug release from various delivery systems [9,10]. In this approach, the alternating anionic and cationic layers form a barrier that impedes drug diffusion. Polyelectrolyte coating of alginate-based hydrogels has been shown to slow down the release of some hydrophilic and hydrophobic drugs [11,12,13,14]. Moreover, the type of coating influences release kinetics differently for each drug, suggesting that release could be fine-tuned depending on the desired application [13]. However, most studies focus on a single type of drug or those with similar physicochemical properties, which restricts the broader applicability of the findings [15,16,17]. Additionally, it remains unclear what types of coatings work best for different types of drugs.

**Figure 1:**
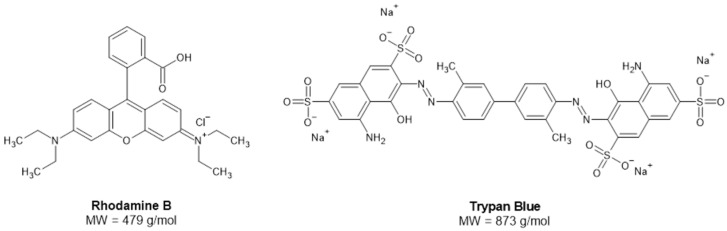
Molecular structures of the model compounds rhodamine B and trypan blue. Rhodamine B is a cationic dye with a positively charged quaternary amine group. Meanwhile, trypan blue has a larger molecular weight and is anionic due to its multiple sulfonate groups. The structures were drawn using ChemSketch (Version 2025).

Electrostatic interactions between the hydrogel matrix and charged drug molecules play a critical role in determining loading efficiency and release kinetics. As alginate carries negatively charged carboxylate groups, its interaction with cationic and anionic compounds is expected to differ substantially, influencing both entrapment and release profiles. Likewise, the efficacy of polyelectrolyte coatings in impeding drug release could also be influenced by the charge of the drug. Understanding these charge-dependent interactions is essential for the rational design of alginate-based delivery systems. To investigate this, we used model hydrophilic compounds: rhodamine B (RhB), a cationic dye, and trypan blue (TB), an anionic dye (Figure 1). Both RhB and TB are widely used in the biomedical field, including as model payloads for drug delivery systems [18,19,20,21]. Systematic evaluation using model compounds with defined ionic characteristics can provide valuable insights into the mechanisms governing drug–hydrogel interactions and release behavior.

In addition to drug characteristics, it is also important to determine the effects of different types of polymer coatings on drug release. Natural polymers such as chitosan (polycation) and alginate (polyanion) are frequently used for biomedical applications due to their intrinsic bioactivity, biocompatibility, and biodegradability. However, they are limited by higher cost and batch variability from inconsistent isolation techniques and/or sources [22,23]. Meanwhile, synthetic polymers such as the polycation polyethyleneimine (PEI) and the polyanion polyacrylic acid (PAA) are generally cheaper and have more predictable properties, but lack intrinsic bioactivity and can accumulate in vivo due to limited biodegradability [22,23]. Aside from origin, these polymers also differ in charge density, which can influence the tightness of the polyelectrolyte layer that is formed [13]. Evaluating the model compounds in different combinations of natural and synthetic polyelectrolyte coatings can aid in optimizing polyelectrolyte-coated alginate hydrogels for various drug delivery applications. 

Therefore, the aim of the present study is to design and evaluate various polyelectrolyte-coated alginate-based hydrogel beads as controlled release systems for both anionic and cationic compounds. Specifically, we aimed to evaluate the effects of novel polyelectrolyte combinations of natural and synthetic polymers on the release of RhB and TB from alginate-based hydrogel beads.

## 2. Results and Discussion

### 2.1. Hydrogel Beads Loaded with Different Model Compounds

**Figure 2:**
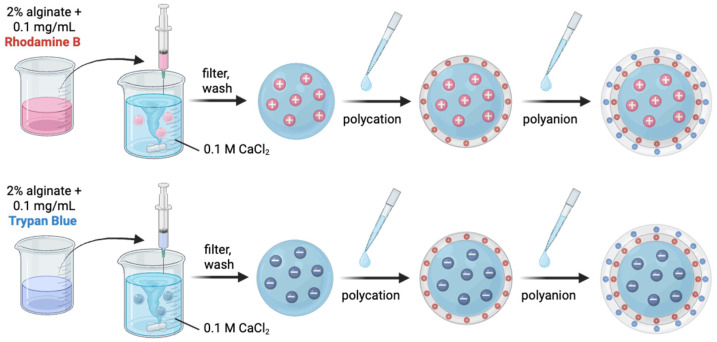
Schematic showing the encapsulation of RhB and TB in polyelectrolyte-coated alginate hydrogel beads. The dyes were mixed with alginate and dropped into a CaCl_2_ solution to create dye-loaded alginate beads. Then, the beads were coated sequentially with polycation (chitosan or PEI) and polyanion (alginate or PAA). Created in BioRender. Marco, K.P. (2026) https://BioRender.com/cxcwy29.

SA-based hydrogels are widely used as carriers for drug delivery and other applications. However, their susceptibility to divalent anions (e.g., citrate and phosphate), limited mechanical strength, inadequate pore barrier properties, and relatively rapid release rates continue to present significant challenges [8]. To address this, we prepared SA hydrogel beads and coated them with various biopolymers and synthetic polymers. SA hydrogel beads were prepared using an ionotropic process wherein, as Ca^2+^ comes in contact with alginate, it forms a gel-like structure. SA forms strong gel matrices through the well-known “egg-box” model, in which calcium ions (Ca^2+^) interact with the guluronic acid blocks of the alginate chains, resulting in a stable hydrogel structure [24]. During preparation, beads were used to entrap two oppositely charged dyes, RhB (positively charged) and TB (negatively charged), as model compounds. When the SA–dye solution was dropped into a CaCl_2_ solution using a syringe, hydrogel beads were formed. The Ca^2+^ ions diffuse into the alginate matrix and create crosslinks between polymer chains, which are essential for the formation of stable microbeads [25]. This leads to the successful formation of spherical plain alginate, alginate–RhB, and alginate–TB hydrogel beads. Figure 2 illustrates the preparation process of alginate hydrogel beads and the entrapment of the dyes, RhB and TB.

### 2.2. Fabrication of Dye-Loaded Hydrogel Beads

**Figure 3:**
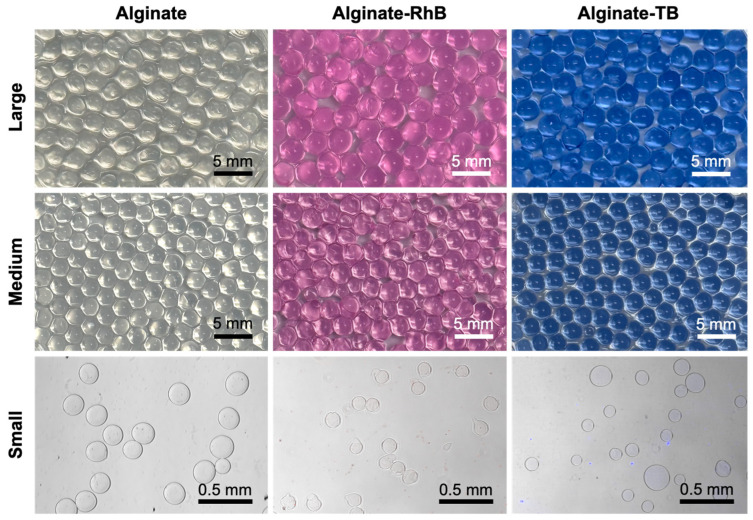
Photographs of different sizes of alginate hydrogel beads. Beads of different sizes were fabricated using varying needle sizes (large—g18; medium—g23; small—g30). Entrapment of RhB and TB is confirmed visually by the pink or blue color of the beads, respectively. Images of small beads were obtained using a microscope. Scale bar = 5 mm (large, medium) or 0.5 mm (small).

Alginate–RhB and alginate–TB hydrogel beads of different sizes (3 mm, 2 mm, and 0.16 mm) were fabricated (Figure 3). The beads were spherical in shape and consistent in size. Hydrogel in bead form is preferred due to its regular size and shape, large surface area, and ability to load a higher amount of drugs [26]. Additionally, dye loading in each of these beads was quantified (Table 1). It was observed that as the size of the beads increased from 0.16 mm to 3 mm, the % drug loading increased from 25.8% to 38.00% for alginate–RhB hydrogel beads. A similar trend was observed in alginate–TB hydrogel beads as well, wherein as the size increased, the % drug loading increased from 20.11% to 29.33%. A plausible explanation for the observed increase in dye loading with increasing bead size is related to greater internal volume and availability of binding/entrapment sites within larger hydrogel beads. As the bead size increases from 0.16 mm to 3 mm, the total polymer content and internal pore volume of the hydrogel bead also increase. Larger beads provide a more extensive three-dimensional polymer network, allowing greater diffusion, an increased number of guluronic acid blocks enabling the formation of more Ca^2+^-mediated crosslinking sites with dye, and retention of dye molecules within the matrix during bead formation. In contrast, smaller beads possess a higher surface-area-to-volume ratio, which can facilitate dye loss to the surrounding CaCl_2_ solution during gelation and washing steps, resulting in lower overall dye loading. Moreover, faster Ca^2+^ diffusion in smaller beads may lead to denser crosslinking near the surface, restricting dye diffusion into the bead core and thereby reducing encapsulation efficiency [27]. Therefore, the combined effects of larger internal volume, enhanced diffusion space, and reduced dye leaching in larger beads likely account for the higher percentage of dye loading observed in both alginate–RhB and alginate–TB hydrogel beads. Sadighian et al. have reported that magnetic-graphene oxide incorporated alginate hydrogel beads showed a loading efficiency of 25.8% for the drug quercetin [2], while Mishra et al. have reported that RhB-nanobeads demonstrated 46.7% RhB loading efficiency [16]. Therefore, the medium-sized beads (2 mm diameter) were selected for succeeding experiments as they have an optimal balance between handling convenience, loading efficiency, and release performance.

**Figure 4:**
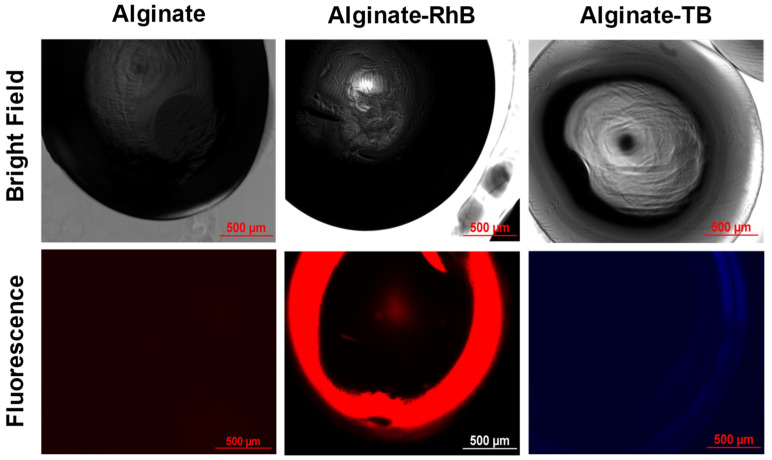
Microscopic analysis of alginate hydrogel beads. Bright-field microscopy shows that the beads are spherical in shape with well-defined surfaces and uniform morphology. Dye loading in alginate–RhB and alginate–TB beads is confirmed through fluorescence of loaded dyes.

Bright-field microscopy of the medium-sized beads demonstrated well-defined surfaces, uniform morphology, and no structural deformities (Figure 4). This suggests the formation of stable beads with intact mechanical integrity. Fluorescence microscopy further confirmed the entrapment of RhB and TB, albeit alginate–TB beads showed weaker fluorescence due to the lower intrinsic fluorescence quantum yield of TB. Overall, these findings led us to proceed with coating the hydrogel beads with different polymers.

### 2.3. Scanning Electron Microscopy

**Figure 5:**
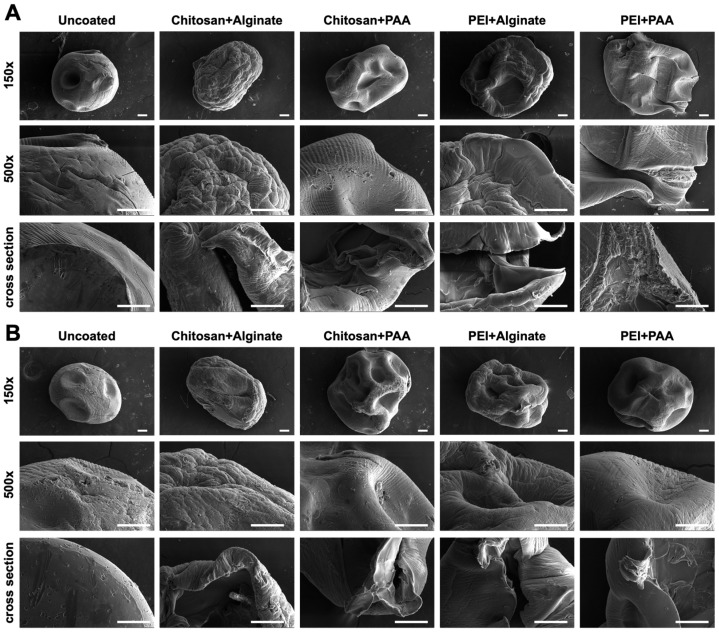
Scanning electron micrographs of uncoated and coated alginate beads. (**A**) Micrographs of alginate–RhB beads. (**B**) Micrographs of alginate–TB beads. All beads significantly shrank after fixation and dehydration. While uncoated beads retained their spherical shape, coated beads appeared collapsed with wrinkled surfaces. Chitosan+alginate and PEI+alginate-coated beads exhibited rougher surfaces and finer wrinkles, while chitosan+PAA and PEI+PAA coatings had smoother surfaces with larger grooves. Micrographs of cut beads show a homogeneous cross-section in uncoated beads and reveal film-like structures in coated beads, demonstrating successful deposition of the polyelectrolyte layers. Scale bar = 100 µm.

Alginate–RhB and alginate–TB beads were coated with four different combinations of polyelectrolyte coatings—chitosan+alginate, chitosan+PAA, PEI+alginate, and PEI+PAA. To confirm successful coating, fixed and dehydrated beads were subjected to scanning electron microscopy (SEM). SEM micrographs revealed that uncoated alginate–RhB beads (Figure 5A) and uncoated alginate–TB beads (Figure 5B) have spherical morphology with mild dimpling. The beads have considerably shrunk from 2 mm to around 0.8 mm in diameter throughout the dehydration process. On higher magnification, the uncoated bead surfaces demonstrated a porous microstructure, suggesting the presence of interconnected pores within the hydrogel matrix. These observations are in accordance with earlier reports [2,28]. In contrast, all coated beads appeared collapsed with wrinkled surfaces. This is likely due to the uneven shrinkage of the alginate hydrogel beads and the polyelectrolyte coatings upon dehydration. As the inner alginate shrank, the structural support of the external coating collapsed, resulting in a deflated appearance. This finding is thus consistent with the coated structure of the alginate beads. Among the different coatings, chitosan+alginate and PEI+alginate resulted in highly wrinkled surfaces with finer grooves in both alginate–RhB and alginate–TB beads. This may be due to the porosity of the external alginate layer. Chitosan+alginate surfaces were particularly rough, which is consistent with the porous structure that is formed when chitosan and alginate are combined to form hydrogels [29]. Meanwhile, chitosan+PAA and PEI+PAA coatings had smoother surfaces with fewer and larger grooves. This suggests the formation of a tightly crosslinked polyelectrolyte complex between the negatively charged PAA and the positively charged chitosan or PEI. The beads were then cross-sectioned to examine their different layers. Uncoated alginate–RhB and alginate–TB beads showed a single homogeneous layer. Meanwhile, shrinkage of the inner alginate bead highlighted the thin external layer in the coated beads, confirming the deposition of polyelectrolyte coatings. Overall, these findings highlight the role of polyelectrolyte coatings in tailoring the surface morphology of alginate hydrogel beads for controlled drug release applications.

**Figure 6:**
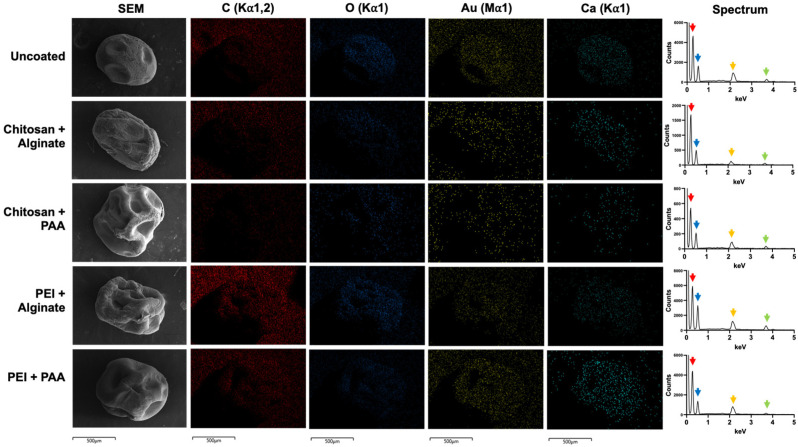
Scanning electron microscopy–energy dispersive spectroscopy analysis. The SEM images of uncoated and coated alginate–TB beads are shown alongside maps of detected elements (C, O, Au, and Ca) at their respective energies. The EDS spectra of each sample revealed major peaks at 0.277 keV (C, red arrow) and 0.525 keV (O, blue arrow). Au, which was used to coat the beads for SEM analysis, was detected as a peak at 2.123 keV (yellow arrow). Lastly, the peak at 3.692 keV represents Ca (green arrow), which was used to crosslink the hydrogel beads. Scale bar = 500 µm.

Energy dispersive spectroscopy (EDS) was then performed to further characterize the different coated alginate hydrogel beads. Alginate–TB beads were used as representatives for this analysis since the type of dye did not appear to affect coating based on the SEM data. As expected, SEM-EDS revealed C and O as the most abundant elements across all uncoated and coated beads (Figure 6). C and O appeared in the EDS spectra as major peaks at their Kα energies of 0.277 keV and 0.525 keV, respectively. Ca was also detected, showing as a peak at its Kα of 3.692 keV, confirming the Ca^2+^-crosslinked alginate structure. The detected Au (Mα 2.123 keV) represents the coating used during sample preparation to improve visualization of beads in SEM. However, there were no notable differences between uncoated and coated beads. Differences in N content could produce insights into the deposition of the polycation layer, as they contain amine groups. However, SEM-EDS is often not sensitive enough for the detection of low-Z elements such as N due to X-ray absorption caused by the Be window of the detector [30]. Additionally, the Kα of N (0.392 keV) can lead to overlapping peaks with those of C and O, which are overwhelmingly abundant in the samples, further complicating analysis.

### 2.4. Swelling Studies

Swelling of SA hydrogel beads is mainly governed by the hydrophilic nature of alginate and the extent of Ca^2+^-mediated crosslinking within the gel matrix [31]. Upon immersion in an aqueous medium, water molecules diffuse into the network, causing polymer chain relaxation and expansion and subsequently promoting drug release. Polyelectrolyte coatings can limit swelling by acting as a diffusion barrier, which is one of their mechanisms of controlling drug release [14].

**Figure 7:**
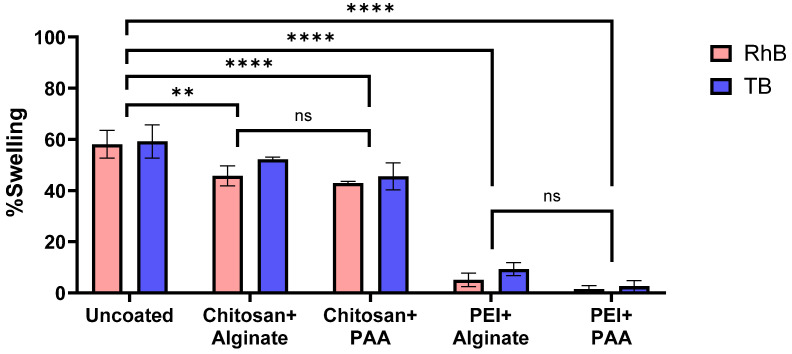
Swelling data of different polyelectrolyte-coated SA hydrogel beads loaded with rhodamine B (RhB) or trypan blue (TB). Uncoated beads had swelling ratios of 58.07–59.20%. Chitosan+alginate and chitosan+PAA coatings moderately reduced swelling to 45.75–52.23% and 42.85–45.52%, respectively. Meanwhile, PEI+alginate and PEI+PAA greatly reduced swelling, with swelling ratios ranging from 5.05–9.26% and 1.50–2.56%, respectively. ns—not significant (*p* > 0.05); ** *p* < 0.01 and **** *p* < 0.0001 using two-way ANOVA (n = 3).

Therefore, we investigated the swelling behavior of different polyelectrolyte-coated SA hydrogel beads (Figure 7). Uncoated alginate–RhB and alginate–TB beads had similar swelling ratios of 58.08 ± 3.39% and 59.20 ± 6.52%, respectively. All coatings, namely chitosan+alginate (*p* = 0.0042), chitosan+PAA (*p* < 0.0001), PEI+alginate (*p* < 0.0001), and PEI+PAA (*p* < 0.0001), significantly reduced swelling compared to uncoated beads. Notably, the effect was largely determined by the polycation. Chitosan+alginate and chitosan+PAA both moderately reduced swelling with no significant difference between the two (*p* = 0.4140). Chitosan+alginate coating reduced swelling of alginate–RhB and alginate–TB beads to 45.75 ± 3.95% and 52.22 ± 0.86%, respectively. Similarly, chitosan+PAA reduced swelling of alginate–RhB and alginate–TB beads to 42.85 ± 0.76% and 45.52 ± 5.26%, respectively. Meanwhile, PEI-based coatings demonstrated greater reductions in swelling. PEI+alginate reduced swelling of alginate–RhB beads to only 5.05 ± 2.66% and alginate–TB beads to 9.26 ± 2.56%. PEI-PAA almost completely inhibited swelling of both alginate–RhB (1.50 ± 1.32%) and alginate–TB (2.56 ± 2.25%) beads. The slightly reduced swelling with PEI-PAA compared to PEI+alginate is possibly due to the more porous surface of the PEI+alginate coating as seen in SEM. However, this difference was not statistically significant (*p* = 0.2620). Overall, the greater reduction in swelling caused by PEI-based coatings suggests a tighter coating formed due to its higher charge density compared to chitosan. This difference in charge density likely affects subsequent coating with the polyanion as well, with PEI having stronger interactions with both alginate and PAA.

Interestingly, the type of dye also influenced the swelling behavior of the alginate beads. RhB-loaded beads generally had significantly lower swelling ratios compared to TB-loaded beads (*p* = 0.0408). This effect was most pronounced with the chitosan+alginate-coated beads (45.75 vs 52.22%). The reason for this is unclear since, to the best of our knowledge, there are currently no studies on the effects of small molecule payloads on the swelling behavior of alginate hydrogels. It is well known that multivalent cations can reduce swelling of alginate hydrogels by increasing crosslinking density [32]. Matyash et al. reported that Na^+^ can restrict the swelling of Ca^2+^-crosslinked alginate hydrogels as well through electrostatic interactions with the COO^−^ groups of alginate despite not affecting crosslinking per se [33]. The cationic RhB could have similarly restricted the expansion and relaxation of alginate polymer chains by electrostatically interacting with COO^−^.

### 2.5. Release of RhB and TB from Hydrogel Beads

**Figure 8:**
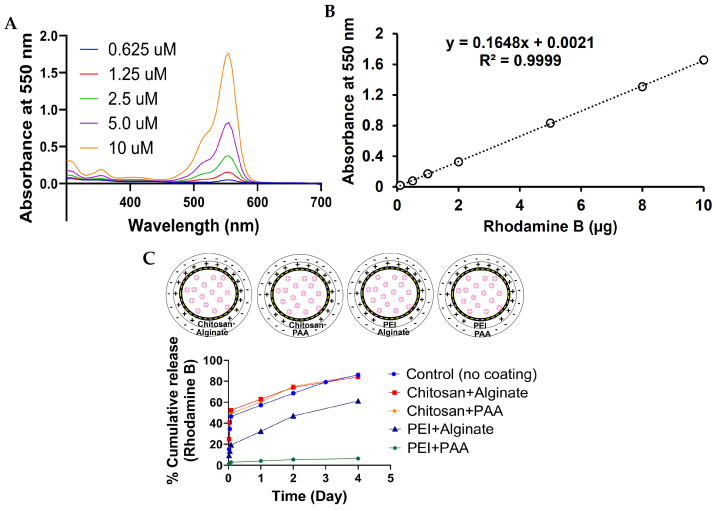
Absorbance spectra of RhB and % cumulative release of RhB from alginate–RhB hydrogel beads. (**A**) UV-Vis spectra of RhB at varying concentrations. (**B**) Calibration plot of RhB. (**C**) RhB release profiles over time. The release of RhB was evaluated over four days for different polymer coatings. Coatings of chitosan+alginate and chitosan+PAA exhibited minimal impact on release, with 84% of the dye released by Day 4. In contrast, PEI+alginate coatings reduced the release to 61%, while PEI+PAA coatings achieved the most significant reduction, with only 6% of the dye released after four days.

The absorbance spectra of RhB at different concentrations were measured (Figure 8A). It was observed that as the concentration of RhB increased from 0.625 µM to 10 µM, a sharp increase in the absorbance peak at 550 nm was observed. Thus, the released RhB from hydrogel beads was evaluated at 550 nm. Figure 8B shows the calibration plot of RhB, and a good linear fit with correlation coefficient R^2^ = 0.9999 was reported. In the case of alginate–RhB, uncoated SA hydrogel beads showed 46% RhB release in 2 h, 57% in 24 h, and 86% release was exhibited in 4 days. This can be explained by the high hydrophilicity and porous structure of the calcium–alginate network, which allows RhB molecules to diffuse readily into the surrounding medium. Burst release within the first 24 h is likely due to the release of surface-associated dye and swelling of hydrogel beads. Similar release behavior was noted for beads coated with chitosan+alginate and chitosan+PAA, as shown in Figure 8C. Although these coatings introduce an additional layer, chitosan itself is hydrophilic and loosely crosslinked with alginate or PAA, resulting in limited diffusion resistance and only marginal restriction of dye release. Overall dye release is also strongly governed by the diffusion of dye molecules through the porous hydrogel matrix and the swelling behavior of the chitosan+alginate beads in the release medium. These diffusion and swelling processes can facilitate dye transport from the hydrogel network, which may reduce the overall impact of electrostatic interactions on delaying the release. As a result, the presence of chitosan did not significantly retard the dye release in the present system. In contrast, beads coated with PEI–alginate exhibited a reduced RhB release of 32% over 24 h and 61% over 4 days. This decrease can be attributed to the strong electrostatic interactions between the positively charged amine groups of PEI and the negatively charged carboxylate groups of SA, leading to a denser and less permeable coating layer [34]. These interactions hinder dye diffusion and slow down the release rate. The slowest release profile, with only 4% RhB release over 24 h and 6% over 4 days, was observed for beads coated with PEI+PAA. This behavior can be explained by the formation of a highly compact and tightly crosslinked polyelectrolyte complex between PEI and PAA, which is consistent with the SEM and swelling studies described above. Additionally, electrostatic interactions between PEI and the cationic RhB molecules may further contribute to dye retention within the hydrogel matrix.

**Figure 9:**
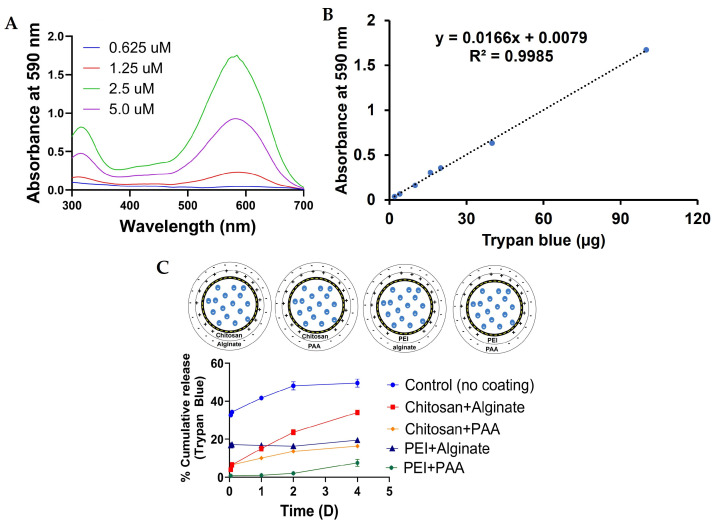
Absorbance spectra of TB, calibration plot, and % cumulative release of TB. (**A**) UV-Vis spectra of TB at varying concentrations. (**B**) Calibration plot of TB. (**C**) TB release profiles over time. The release of TB was significantly reduced by all polymer coatings compared to uncoated hydrogels, which released 49% of the dye over four days. Coatings of chitosan+alginate, PEI+alginate, chitosan+PAA, and PEI+PAA progressively decreased dye release to 34%, 19%, 16%, and 7%, respectively, demonstrating the effectiveness of these coatings in controlling dye diffusion.

The absorbance spectra of TB at different concentrations were measured (Figure 9A). It was observed that as the concentration of TB increased from 0.625 µM to 5 µM, a sharp increase in the absorbance peak at 590 nm was observed. Thus, released TB from alginate–TB was monitored at 590 nm. Figure 9B shows the calibration plot of TB, and a good linear fit with correlation coefficient R^2^ = 0.9985 was observed. In the case of uncoated alginate–TB hydrogel beads, a cumulative release of 41% TB over 24 h and 49% over 4 days were observed (Figure 9C). This relatively higher release is attributed to the hydrophilic and porous nature of the calcium–alginate network, which allows TB molecules to diffuse readily into the surrounding medium. Upon coating the beads with additional polymer layers, a progressive reduction in dye release was observed. Beads coated with chitosan+alginate exhibited a reduced release of 15% and 34% over 24 h and 4 days, respectively, which can be attributed to the formation of a polyelectrolyte complex between positively charged chitosan and negatively charged alginate. This coating increases the diffusion path length and partially restricts TB transport. Further, a reduction in release was observed for PEI+alginate-coated beads (16% over 24 h and 19% over 4 days), likely due to stronger electrostatic interactions between the highly cationic PEI and alginate, resulting in a denser and less permeable coating layer. Similarly, beads coated with chitosan+PAA (10% over 24 h and 16% over 4 days) exhibited enhanced diffusion resistance due to increased crosslinking and polymer entanglement within the coating. The slowest TB release (1% over 24 h and 7% over 4 days) was observed for beads coated with PEI+PAA, which can be attributed to the formation of a highly compact and tightly crosslinked polyelectrolyte complex. Overall, these results demonstrate that surface modification using different polymer combinations effectively controls RhB and TB diffusion, with PEI-based coatings, particularly PEI+PAA, providing the greatest resistance to dye release and enabling sustained release behavior.

In summary, PEI-PAA emerged as the best candidate for coating SA hydrogel beads and slow release of model dyes. The differences in release behavior between RhB and TB arise from variations in their physicochemical properties and their interactions with the hydrogel matrix and surface coatings. First, the molecular size of dyes plays an important role, as RhB is a relatively small molecule, which allows it to diffuse more easily through the pores of the alginate hydrogel network. In contrast, TB has a larger molecular size and more complex structure, which restricts its mobility within the hydrogel and results in slower release. Second, the charge characteristics of the dyes significantly influence their release. Alginate possesses negatively charged carboxylate groups, which can interact electrostatically with the positively charged RhB and retard diffusion out of the beads. RhB also experiences electrostatic attraction with the anionic alginate and PAA coatings, but can still diffuse out depending on pore size and coating permeability. In contrast, TB, being negatively charged, exhibits weaker interaction with alginate in the bead matrix. It forms strong electrostatic interactions with cationic coatings such as chitosan and PEI through its negatively charged sulfonate groups, leading to tighter binding and reduced diffusion. Additionally, polymers such as PAA primarily interact through hydrogen bonding and physical entrapment rather than strong electrostatic interactions, which may further affect the release behavior. In summary, the combined effects of molecular size, charge, hydrophilicity, and interaction strength with the hydrogel and coatings account for the observed differences in release profiles of RhB and TB.

Meanwhile, the differences in dye release behavior between the different coatings could be explained by differences in characteristics and electrostatic interactions between the polyelectrolytes. The synthetic polymers PEI and PAA have higher charge densities than alginate and chitosan, which results in a tighter coating that impedes dye release [13]. This is supported by the results of SEM and swelling studies. The surface morphology of the beads was largely influenced by the external polyanion layer, wherein PAA resulted in a less porous surface compared to alginate. Additionally, swelling was mainly influenced by the polycation layer, with PEI greatly reducing swelling compared to chitosan. PEI+PAA has the combined advantages of reduced swelling and a less porous surface, leading overall to the greatest retardation of dye release.

The findings from this study can be used to improve alginate-based hydrogel delivery systems for various drugs. In particular, the variability in release rates for TB with the different polyelectrolyte coatings suggests their potential for fine-tuning the drug release of other hydrophilic anionic drugs with a similar molecular weight. For instance, suramin is a TB-like drug that is being explored for various therapeutic applications, including wound healing, prevention of vascular restenosis, and treatment of solid tumors [35,36,37,38,39]. However, its hydrophilicity and rapid clearance limit systemic use; hence, delivery systems, including locally administered hydrogels, are being developed [35,37,38,39]. The coated alginate hydrogel beads in this study are therefore promising carriers for suramin and other anionic drugs, and the combination of polyelectrolyte coatings used can be tuned to the desired release rate.

This study has certain limitations, such as being limited to in vitro experiments under controlled laboratory conditions. Release studies were performed at room temperature, with a buffer solution that mimics the physiological pH of biological fluids, through serial replacements of the extraction medium to maintain sink conditions. However, the performance of the polymer-coated hydrogels in physiological environments, where factors such as enzymes, ionic strength, and dynamic fluid flow are present, remains to be evaluated. There is a need to evaluate the potential cytotoxicity of PEI-based coatings at higher concentrations before biomedical applications can be considered. The long-term structural stability, degradation behavior, and mechanical integrity of the coated hydrogel beads need to be investigated, which are critical parameters for sustained drug delivery systems. Also, release studies were conducted under a single set of conditions. The influence of pH, ionic strength, and temperature on release kinetics was not explored. Sink conditions were maintained by ensuring that the total dye concentration used in release studies was much lower than its solubility in the release medium. However, larger amounts of extraction medium can be used to confirm that dye saturation did not influence release behavior. Moreover, the release studies were performed with a few time points for up to four days. In future studies, release kinetics can be more extensively characterized with more time points and for longer durations (at least 14 days). This will also enable fitting the release data with mathematical models to gain mechanistic insights into the processes that govern drug release in these hydrogel systems. Finally, FTIR analysis of coated beads could be used to investigate the dye–alginate and alginate–polyelectrolyte interactions within the hydrogel beads and potentially explain the differences observed between the different hydrogel systems. Addressing these limitations in future studies would strengthen the translational potential of polymer-coated alginate hydrogels for controlled drug delivery applications.

## 3. Conclusions

To enhance the applicability of alginate-based hydrogels, the beads were surface-modified with various polymers and employed as carriers for loading the model dyes RhB and TB. The uncoated alginate beads exhibited dye loading efficiencies of 38% for RhB and 29.33% for TB. Polyelectrolyte coating using different combinations of natural and synthetic polymers was confirmed via SEM, with PAA-based coatings showing less porous surfaces than alginate-based coatings. Swelling studies revealed PEI-based coatings as effective diffusion barriers. Surface modification using different polymer combinations effectively modulated the diffusion and release behavior of both dyes. Among the various coatings studied, PEI+PAA provided the greatest resistance to dye release, resulting in a markedly sustained release profile. Overall, PEI+PAA emerged as the most effective coating for alginate hydrogel beads in achieving controlled and prolonged release of the model dyes. These findings demonstrate that polymer-coated alginate hydrogels offer a versatile platform for regulating the release of charged molecules, highlighting their potential utility in controlled drug delivery applications. Furthermore, optimization of polymer combinations may enable fine-tuning of release kinetics to meet the requirements of specific therapeutic applications. Future studies can include more detailed characterization of the polyelectrolyte-coated hydrogel beads, including more extensive drug release studies with fitting to mathematical models and FTIR analysis of interactions between the different components within the hydrogel systems.

## 4. Materials and Methods

### 4.1. Materials

Sodium alginate (CAS-No. 9005-38-3, 12–40 kDa), CaCl_2_ (>97%), PBS at pH 7.4, sodium citrate (ACS reagent, ≥99.0%), chitosan (50–190 kDa), polyacrylic acid (PAA) (2 kDa), polyethyleneimine (PEI) (branched, 25 kDa), rhodamine B (RhB) and trypan blue (TB) were purchased from Sigma-Aldrich (St. Louis, MO, USA). All chemicals were used without further purification unless otherwise mentioned.

### 4.2. Preparation of Beads

SA hydrogel beads were prepared by dissolving SA (2% *w*/*v*) in MilliQ water and adding either RhB (0.1 mg/mL) or TB (0.1 mg/mL) dye to the solution. For this, 2 g SA powder was added to 100 ml MilliQ water and stirred at 100 rpm for 30 min. Once fully dissolved, RhB or TB (10 mg) was added and stirred again for 15 min. For the synthesis of alginate–RhB or alginate–TB hydrogel beads, the alginate–dye solution was loaded into a syringe with different needle gauges (g18, g23, and g30) and dropped into a 100 mM calcium chloride (CaCl_2_) solution with gentle stirring at 100 rpm. For the small beads (g30), the alginate–dye solution was electrosprayed into the CaCl_2_ solution at a flow rate of 0.05 mL/min and voltage of 7.0 kV using a standard electrospray system (Spraybase, Cambridge, MA, USA). The beads were allowed to cure in the CaCl_2_ solution for 30 min under stirring. After curing, the hydrogel beads were filtered and washed three times with Milli Q water to remove unbound excess dye. Recovered hydrogel beads were stored at 4 °C for further experiments.

### 4.3. Characterization

A total of 200 mg of beads was carefully weighed and counted to determine bead size distribution. Photographs of the beads were taken, and their morphology was assessed using optical microscopy (Nikon Eclipse TsR microscope, Nikon, Melville, NY, USA). Images were analyzed to evaluate bead uniformity and size.

### 4.4. Coating with Different Polymers

Coating of the beads was performed by immersing the beads in 1% (*w*/*v*) solutions of different polymers (alginate, chitosan, PAA, and PEI) for 1 min. A total of 8 types of coated beads were prepared, with 4 types of coatings for each of the dye-loaded beads, as shown in Table 2. To prepare the 1% chitosan solution, 1 g of chitosan was dissolved in 1% acetic acid with constant stirring at 150 rpm on a magnetic stirrer for 5 h. For PAA, 1% solution was prepared by dissolving 1 g of PAA in water with stirring at 150 rpm using a magnetic stirrer for 1 h. PEI solution (1%) was prepared by using its stock solution, and the pH of the resulting solution was adjusted to 7.0. Then, the alginate beads were coated layer-by-layer with the polymers. First, 1 mL of polycation solution was added to dye-loaded alginate beads and incubated with gentle shaking for 1 min. This allowed the polycation to attach to the anionic alginate bead surface. The supernatant was then removed, and the beads were washed with 1 mL Milli-Q water for 1 min. Then, the process was repeated with 1 mL of polyanion solution.

### 4.5. SEM-EDS

Uncoated and coated alginate–RhB and alginate–TB beads were examined using scanning electron microscopy. Beads were fixed in 2.5% glutaraldehyde and subjected to gradient dehydration with ethanol. To examine cross-sectional surfaces, dehydrated beads were sectioned using a scalpel prior to air drying. Dried samples were then sputter-coated with a 10 nm layer of gold and scanned using the FEI Helios NanoLab 660 FIB-SEM (Thermo Fisher Scientific, Waltham, MA, USA). The samples were then scanned using SEM-EDS at a voltage of 15 kV.

### 4.6. Swelling Studies

To study the swelling property of hydrogel beads, 200 mg of beads were placed in 5 mL Milli-Q water for 24 h. Afterwards, the beads were sieved, collected, and weighed. Using the following equation, the % swelling was calculated:

% Swelling = ((Weight_(24h)_ − Weight_(initial)_)/Weight_(initial)_) × 100% (1)

where weight_(24h)_ is the weight recorded at 24 h and weight_(initial)_ is the initial weight of the beads.

### 4.7. Dye Loading Determination

Dye loading efficiency (%) in the beads was determined by an extraction method followed by UV–Vis spectrophotometric analysis. Briefly, 100 mg of beads was dissolved in 1 mL of phosphate buffer and sonicated for 30 min at room temperature to ensure complete extraction of the loaded dye. Then, the suspension was analyzed using a UV–Vis spectrophotometer at 550 nm for RhB and 590 nm for TB. The concentration of dye was calculated using a standard calibration curve. Dye loading (%) was calculated using the following equation:

Dye loading (%) = (Weight of dye loaded in the beads/weight of beads) × 100(2)

### 4.8. Release of Loaded Compound

Release studies were conducted by immersing 200 mg of beads in 1 mL of phosphate-buffered saline (PBS, pH 7.4) at room temperature in triplicate, as previously described [40,41]. The PBS solution was carefully withdrawn using a micropipette and replaced with 1 mL of fresh PBS at predetermined time points (e.g., 1 h, 2 h, 1 day, and 4 days). The collected samples were analyzed using a Cary 60 SW UV-Vis spectrophotometer (Agilent, Santa Clara, CA, USA). The absorbance of RhB (λ_max_ = 550 nm) or TB (λ_max_ = 590 nm) was measured. A standard plot of RhB or TB was plotted using different concentrations and used to quantify the released dye. The release profile was determined by monitoring the changes in absorbance over cumulative dye release calculated using Equation (3):

Cumulative dye release (%) = (Qt/Q_0_) × 100 (3)

where Qt represents the amount of dye released at given time t, and Q_0_ is the total amount of dye loaded in the beads.

### 4.9. Statistical Analysis

All studies were conducted in triplicate (n = 3) or three independent experiments. The data were presented as mean ± SD. GraphPad Prism, version 10.1.2 software (GraphPad, San Diego, CA, USA) was used to perform all statistical analyses. A one-way analysis of variance (ANOVA) or two-way ANOVA was used, and differences were considered significant at *p* < 0.05.

## Figures and Tables

**Table 1:** % Loading of model compound in the hydrogel beads of various sizes.

	Alginate–RhB			Alginate–TB		
	Large	Medium	Small	Large	Medium	Small
**Size (mm)**	3	2	0.16	3	2	0.16
**% Dye loading**	38.00	31.87	25.8	29.33	23.56	20.11

**Table 2:** Types of polyelectrolyte-coated alginate-based hydrogel beads used in the study. Alginate beads containing the dye were coated sequentially with polycation and polyanion.

Dye	Polycation	Polyanion
RhB	Chitosan	Alginate
	Chitosan	PAA
	PEI	Alginate
	PEI	PAA
TB	Chitosan	Alginate
	Chitosan	PAA
	PEI	Alginate
	PEI	PAA

## Data Availability

The original contributions presented in this study are included in the article. Further inquiries can be directed to the corresponding author.
